# Low loss and high performance interconnection between standard single-mode fiber and antiresonant hollow-core fiber

**DOI:** 10.1038/s41598-021-88065-2

**Published:** 2021-04-22

**Authors:** Dmytro Suslov, Matěj Komanec, Eric R. Numkam Fokoua, Daniel Dousek, Ailing Zhong, Stanislav Zvánovec, Thomas D. Bradley, Francesco Poletti, David J. Richardson, Radan Slavík

**Affiliations:** 1 Department of Electromagnetic Field, Czech Technical University in Prague, Technická 1902/2, 166 27 Prague 6, Czech Republic; 2 Optoelectronics Research Centre, University of Southampton, Southampton, SO17 1BJ UK

**Keywords:** Applied optics, Fibre optics and optical communications

## Abstract

We demonstrate halving the record-low loss of interconnection between a nested antiresonant nodeless type hollow-core fiber (NANF) and standard single-mode fiber (SMF). The achieved interconnection loss of 0.15 dB is only 0.07 dB above the theoretically-expected minimum loss. We also optimized the interconnection in terms of unwanted cross-coupling into the higher-order modes of the NANF. We achieved cross-coupling as low as −35 dB into the LP$_{11}$ mode (the lowest-loss higher-order mode and thus the most important to eliminate). With the help of simulations, we show that the measured LP$_{11}$ mode coupling is most likely limited by the slightly imperfect symmetry of the manufactured NANF. The coupling cross-talk into the highly-lossy LP$_{02}$ mode ($>2000$ dB/km in our fiber) was measured to be below −22 dB. Furthermore, we show experimentally that the anti-reflective coating applied to the interconnect interface reduces the insertion loss by 0.15 dB while simultaneously reducing the back-reflection below −40 dB over a 60 nm bandwidth. Finally, we also demonstrated an alternative mode-field adapter to adapt the mode-field size between SMF and NANF, based on thermally-expanded core fibers. This approach enabled us to achieve an interconnection loss of 0.21 dB and cross-coupling of −35 dB into the LP$_{11}$ mode.

## Introduction

Hollow core fibers (HCFs) have been reported to reduce the attenuation from 1.3 dB/km a year ago^1^ to the current state-of-the-art of 0.28 dB/km^2^, making the prospect of obtaining an optical fiber with attenuation below that of solid silica single-mode fiber (SMF) a distinct possibility in the near future. The latest three low-loss records in HCFs (1.3 dB/km^1^, 0.65 dB/km^3^, and 0.28 dB/km^2^) were reported in the Nested Antiresonant Nodeless Fiber (NANF), which therefore emerges as the most promising geometry in the quest for lower losses than the SMF. Besides this potential for ultra-low attenuation, HCFs have a range of additional advantages including low latency of propagation^4^, low thermal sensitivity of latency as well as accumulated phase^5^, low nonlinearity^6,7^, high damage threshold^8^, etc., making them of interest in a wide range of applications. To fully benefit from these advantages, however, an efficient low-loss and low-back-reflection interconnection between HCFs and mainstream SMFs is needed.

Recently, we demonstrated a new permanent, low-loss SMF-HCF interconnection technique based on gluing rather than splicing^9^. Fiber gluing is a widely-used process in demanding and cost-sensitive applications including telecoms, e.g., for pigtailing of planar lightwave splitters^10^. As gluing does not require any heating of the fibers (as opposed to fusion splicing), there is no deformation to the HCF micro-structure, which otherwise increases the interconnection loss. Further, no heating means that an optical coating can be applied in between the HCF and SMF. When anti-reflective (AR) coating is deposited, it significantly reduces the unwanted 3.5% back-reflection occurring at the glass-air interface in between the air core of the HCF and the solid silica glass core of the SMF. In addition to reducing the back-reflection, it also lowers the insertion loss (by up to 3.5% that would otherwise be lost in the back-reflection). The most important aspect, however, that must be addressed to achieve low-loss SMF-HCF interconnection, is to adapt the mode-field diameter, which at 1550 nm is typically $10.4\,\upmu \hbox {m}$ for the SMF and $>20\,\upmu \hbox {m}$ for low-attenuation HCF^9^.

In^9^, we inserted a short segment of graded-index multi-mode fiber (GRIN) in between the SMF and HCF, which served as a lens that adapted the mode field size. When complemented with the AR coating, an HCF-SMF insertion loss (IL) as low as 0.3 dB and back-reflection below −30 dB were achieved. This was demonstrated with a photonic bandgap type of HCF, which was the state-of-the-art in terms of attenuation before the NANF emerged. These two types of HCFs are fundamentally different in their guiding mechanism (photonic bandgap guiding versus antiresonant effect guiding) as well as structure (delicate photonic bandgap with a large number of thin glass membranes versus a small number of tubes). As the antiresonant HCFs emerged as a low-attenuation solution only recently, there is only a limited number of reports on their interconnection with SMF. To the best of our knowledge, the lowest interconnection loss reported is 0.5 dB for simple SMF-NANF interconnection^11^ or 1.5 dB for a more complex device (1 $\times$ 2 splitter^12^).

Although the lowest-loss value reported for a HCF-SMF interconnection of 0.3 dB is acceptable for a wide range of applications, it is 0.17 dB higher than expected from simulations^9^. Additionally, there are no data on how much of the light is coupled into the HCF’s higher-order modes (HOMs) in these low-loss interconnections, and (even more importantly) how and how much this can be suppressed. From the practical point of view, loss lower than 0.3 dB will be of interest in high-power applications and laser resonators. Lower unwanted coupling into HOMs is of interest in applications sensitive to modal noise, e.g., interferometry^13^, precise time^14^, frequency transfer^15^ and even telecommunications^16,17^.

In this paper, we show a record low-loss SMF-NANF interconnection of 0.15 dB (which increases slightly to 0.16 dB when permanently glued), which is only 0.07 dB higher than the theoretically predicted minimum loss of 0.08 dB (calculated from the mode-field overlap between the field in NANF and a Gaussian field profile). This represents over 2 times improvement from the previous result in which the interconnection loss of 0.30 dB was 0.17 dB above the theoretical limit^9^. This improvement is thanks to the use of a mode-field adapter that is better matched to the mode of the HCF used. Simultaneously, we report a low-back-reflection level of −40 dB over 60 nm bandwidth thanks to a high-performance AR coating. Furthermore, we calculated and measured the coupling into the HOMs and show that: (1) the coupling magnitude to the LP$_{11}$ mode was measured to be below −35 dB (calculated value: −41 dB) and that it is likely to be limited by the symmetry of the fabricated NANF, and (2) coupling magnitude to the LP$_{02}$ mode was measured to be below −22 dB (calculated value: −24 dB). In addition to the GRIN-based mode field adaptation, we also present a new configuration based on thermally-expanded core fibers (TECs), showing experimentally a NANF-SMF interconnection loss of 0.21 dB.

## Simulations of NANF mode field profile and higher-order modes

**Figure 1:**
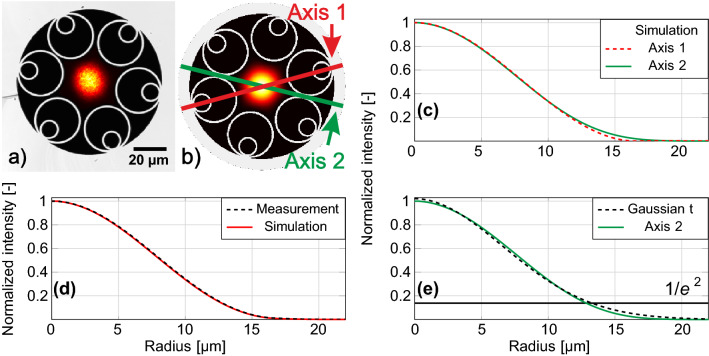
(**a**) Measured mode field distribution at the output of NANF overlaid with the captured image of the NANF core area. (**b**) Simulated fundamental mode field distribution overlaid with the NANF microstructure, extracted from the used NANF end-face photograph. (**c**) Mode field profiles from (**b**) along the two principal axes shown in (**b**). (**d**) Averaged axis 1 and 2 mode field profiles from measurement and simulation. (**e**) Mode field profile from (**c**) and its Gaussian fit.

NANF HCF used in our experiment is of the same geometry as reported in^1^, operating in the 2nd antiresonant window at 1550 nm. It has six large tubes with smaller tubes placed inside them, see Fig. 1a. The measured core diameter is $32.5\,\upmu \hbox {m}$.

Firstly, we measured the field profile of light at the NANF output. The result together with an image of the used NANF end-face are shown in Fig. 1a. Subsequently, we simulated the fundamental mode-field profile of the NANF used in our experiments (obtained using the fiber end-face image shown in Fig. 1a and technique described in detail in^18^) using COMSOL Multiphysics. Both simulated and measured mode field profiles show that NANF mode is not circularly symmetric—it actually has a six-fold symmetry, following the symmetry of the fiber microstructure. This is also visualized in Fig. 1c, where the mode field profile is plotted along the two principal axes (that are shown in Fig. 1b).

Let us consider light coupling between the NANF fundamental mode and a Gaussian-profile beam. A priori, we expect the mismatch between the circular symmetry of the Gaussian beam and the six-fold symmetry of the NANF fundamental mode to impose a fundamental limit on the achievable interconnection loss. However, the symmetry is not the only limiting factor, as we can see by analyzing the data shown in Fig. 1e, where one of the mode field profiles from Fig. 1c is fitted with a Gaussian profile. Clearly, the NANF mode field profile is not Gaussian, which is especially visible in the mode field tails, where the Gaussian profile vanishes more slowly than the profile of the NANF fundamental mode. Both of these phenomena (the non-circular symmetry and non-Gaussian mode field profile) contribute to: (1) the minimum-achievable coupling loss between a Gaussian-profile beam and the fundamental mode of NANF, and (2) coupling into HOMs. Theoretical analysis (calculating overlap integrals between the Gaussian mode and the NANF modes) shows that the minimum coupling loss from a Gaussian beam into NANF fundamental mode is 0.08 dB, with simultaneous coupling into LP$_{02}$ mode (essentially a cross-talk) of −24 dB. Coupling into the LP$_{11}$ mode is −41 dB. The LP$_{11}$ coupling occurs due to the small deviations from the ideal symmetry (fabrication errors) of the manufactured NANF structure, this coupling would ideally be zero thanks to the different symmetries of the fundamental mode and the LP$_{11}$ mode. The attenuation of the fundamental, LP$_{11}$, and LP$_{02}$ modes (limited by confinement loss) is calculated to be $\sim$ 0.6 dB/km, $\sim$ 35 dB/km and $\sim$ 2100 dB/km, respectively.

## Mode field adaptation

There are two aspects to be addressed to obtain ultra-low-loss interconnection between NANF and standard SMF: (1) The differences in mode field profile between the NANF and SMF-28 and (2) the Fresnel losses and back-reflection caused by the difference in refractive indices at a glass–air interface (about 3.5% for a silica glass–air interface).

As for the mode field adaptation, we would ideally need to transform the mode field profile and size of the SMF to match those of the NANF. As discussed earlier, transforming the mode field profile is challenging due to the different symmetry (circular for SMF and six-fold for NANF) as well as the slightly different field profiles: both SMF and NANF modes are almost Gaussian, but as we have shown in Fig. 1e they do not fit the Gaussian profile perfectly. Thus, we attempt to match the mode field size only, accepting this will limit the minimum-achievable coupling loss. As shown in our theoretical analysis, this limitation is expected to be 0.08 dB (provided we generate a perfectly-Gaussian mode field profile of optimum size).

To enlarge the mode field diameter (MFD) of SMF $(10.4\,\upmu \hbox {m})$ to that of our NANF $(\sim 24\,\upmu \hbox {m})$, we use here two approaches: the first one uses commercially available graded-index multi-mode fiber (GRIN) as mode field adapters (MFA), the other one uses SMF-based TEC mode field adapters.

### GRIN and TEC fiber based MFAs

An optical signal that propagates from SMF to GRIN enlarges and shrinks its MFD in a periodic/sinusoidal fashion along the length of the GRIN (one period is referred to as ‘one pitch’). It is therefore possible to achieve the desired MFD by controlling the length of the GRIN segment. The largest MFD is obtained when the GRIN length is at 1/4 of the pitch, which is also the length at which the output beam is collimated. In our experiment, we splice the GRIN fiber to SMF-28, put it into a single-channel glass fiber array (FA) and polish it to the desired length, which depends on the required MFD. We described this technique in detail in our previous publication^9^.

**Figure 2:**
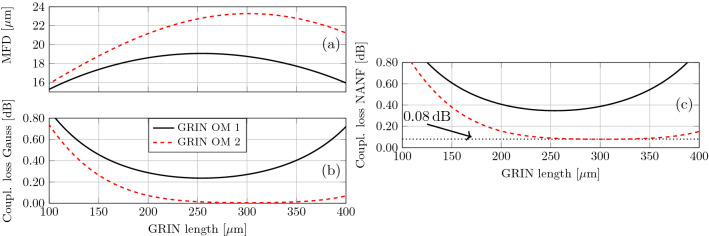
(**a**) Calculated MFD of OM1 (solid line) and OM2 (dashed line) type GRIN as a function of its length. (**b**) Coupling loss between Gaussian mode with MFD of $24.1\,\upmu \hbox {m}$ and output of GRIN mode field adapter shown in (**a**). (**c**) Coupling loss between NANF fundamental mode and output of GRIN mode field adapter shown in (**a**).

We consider commercially available GRIN fibers of type OM1 (core diameter $62.5\,\upmu \hbox {m}$, numerical aperture NA = 0.275) and OM2 (core diameter $50\,\upmu \hbox {m}$, NA = 0.20). We used BeamProp software to model propagation through the GRIN fibers (OM1 and OM2) to get an estimate of the 1/4 pitch length as well as the output MFD. The modelling results for GRIN length in the vicinity of the 1/4 pitch (which is the region of our interest, as we show later) are shown in Fig. 2a. MFD is calculated at the $1/e^2$ of the intensity profile. We see that OM1 type GRIN provides a MFD up to $19.1\,\upmu \hbox {m}$ (at 1/4 pitch length of $260\,\upmu \hbox {m}$), while OM2 type GRIN offers a MFD up to $23.2\,\upmu \hbox {m}$ (at 1/4 pitch length of $300\,\upmu \hbox {m}$). The main reason OM2 provides larger MFD as compared to OM1 is its lower NA. Fig. 2b shows coupling loss between the mode generated by the GRIN MFA (which we measured in the near-field to be Gaussian) and a Gaussian mode with $24.1\,\upmu \hbox {m}$ MFD (MFD value obtained by fitting the NANF mode with a Gaussian profile). We see that OM2 should provide close to 0 dB loss for coupling into a Gaussian mode of $24.1\,\upmu \hbox {m}$ MFD for a GRIN length of 250–350$\,\upmu \hbox {m}$. Fig. 2c shows the coupling loss between the mode generated by the GRIN MFA (considered to be Gaussian) and mode of our NANF (which includes the loss due to the mode field diameter mismatch (shown in Fig. 2b) together with loss due to NANF’s 6-fold symmetry and its slightly non-Gaussian field profile) with the earlier-discussed limit of 0.08 dB. However, MFD is not the only parameter of the beam leaving GRIN fiber. Unless it is at 1/4 pitch, the beam is converging (focusing) or diverging, introducing phase curvature across the mode profile. This effect is not considered in our simulations.

**Figure 3:**
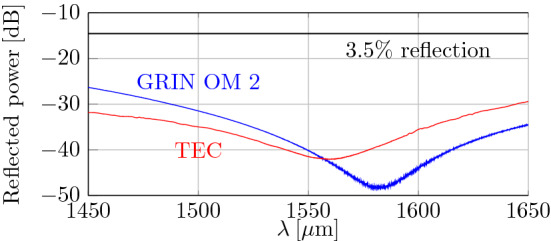
Measured back-reflection of SMF-GRIN and SMF-TEC MFAs as a function of wavelength.

To overcome Fresnel losses, we use an AR coating applied on to the polished GRIN surface^9^. To deposit this coating, particular care must be given to the coating deposition temperature, as the GRIN is glued inside the FA. Glue may not withstand high temperatures used for standard thin-film coating procedures and lower deposition temperature may compromise the coating quality. This is why in our previous report, we achieved a back-reflection level of −30 dB only. Although this level of back-reflection does not degrade the interconnection loss (−30 dB back-reflection corresponds to 0.1% of transmission loss, which is negligible as compared to the expected insertion loss of 0.08 dB (1.8%) transmission loss), better back-reflection suppression would be of interest in many applications. Here, we improved the control of the AR deposition process, achieving more than 10 dB better back-reflection suppression. The AR coating is a 4-layer TiO$_2$/SiO$_2$ design, which allows suppression of back-reflection by up to 48 dB, with >40 dB suppression over a 60 nm bandwidth (1550–1610 nm) (Fig. 3).

TECs are commercially-available and typically provide 2-3 times magnification of the mode field size of standard SMF. We purchased TECs with mode field diameters of 20, 24, and $26\,\upmu \hbox {m}$ and a deposited AR coating of 40-dB back-reflection-level at 1550 nm as shown in Fig. 3.

## SMF-NANF interconnection characterization

### Measurement setup

As NANF can support the guidance of HOMs, we need to be cautious when measuring a single SMF-NANF interconnection to accurately characterize the coupling between the SMF and the NANF fundamental mode. This is because measuring power directly at the NANF output includes contributions from all the propagating modes and HOMs can carry an appreciable fraction of the total optical power.

One way to measure coupling between the SMF and NANF fundamental mode is to use a long length of NANF in which all HOMs are attenuated (thanks to the relatively high differential loss between the NANF fundamental mode and HOMs), obtaining only the fundamental mode power at the NANF output.

**Figure 4:**
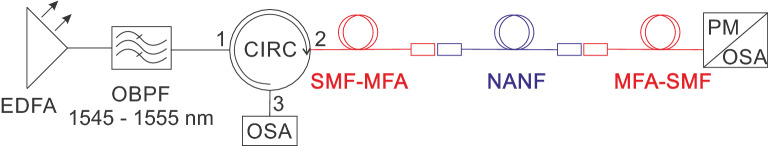
Setup used for insertion loss and back-reflection measurement of SMF-NANF-SMF interconnect for GRIN/TEC MFA pairs.

We chose another approach, using a short NANF (10 m) and performing interconnection with the SMF at both ends using a pair of identical MFAs (Fig. 4). This method allows for accurate coupling loss measurement between the fundamental modes of the two fibers^9^ and also allows for estimation of the coupling efficiency into HOMs, which is not possible using a single interconnection and a long length NANF (HOMs will already be attenuated and no interference pattern will be visible). This approach provides additional means of characterization of the interconnection performance.

We took a set of GRIN and TEC MFA pairs with various mode field sizes. For each MFA pair, we aligned them with a 10 m long NANF sample and measured the total insertion loss as shown in Fig. 4. We used an Erbium-doped fiber amplifier (EDFA) operated in automatic power control mode as a broadband light source (Keyopsys KPS-BT2-C-10-LN-SA). We chose this source as it is broadband and unpolarized, avoiding effects of polarization dependent loss or interference during the alignment. The EDFA signal was filtered with a 10 nm wide optical band-pass filter (OBPF) (1545–1555 nm). The output of OBPF was then passed through a circulator (CIRC) to the input of the first MFA. The MFA output was precisely aligned with NANF using a 5-axis micropositioning stage (Thorlabs NanoMax MAX313D/M with pitch and yaw tilt platform APY002/M). Port 3 of CIRC enabled measurement of the back-reflection. The NANF output was aligned in a similar fashion to the second MFA.

There are two important aspects of our measurement we would like to emphasise: firstly, the use of the 5-axis stages that enables pitch and yaw alignment. Besides angular misalignment compensation, it also allows for compensation of small imperfections in the NANF cleave angle, which was always below 1$^{\circ }$ in our experiments. Secondly, the accurate calibration of transmitted power for accurate insertion loss measurement. For this, we firstly measured the transmitted power using an SMF patchcord and considered it as our reference (zero loss) value. Subsequently, we cut this patchcord in the middle and spliced the two MFAs in. Finally, we inserted the NANF sample in between the two MFAs and measure the total insertion loss of the SMF-NANF-SMF interconnections. Loss of a single SMF-NANF interconnection is then given as half of this value. This, however, means our loss measurement includes two SMF-SMF splices, which we do not account for in our analysis and which may cause up to 0.01  dB loss each. Although this makes for a slightly overestimated loss value, this is, however, at the level of accuracy of our power measurement (0.01 dB) and is thus considered negligible.

The output signal was captured using either a power meter (PM, Thorlabs S154C, to characterize insertion loss) or an optical spectrum analyzer (OSA, Yokogawa AQ6370C, to characterize back-reflection and HOM interference). This approach allows us to repeatably achieve the insertion loss of 0.15 dB within ± 0.01 dB accuracy.

As analyzed in simulations, we expect some level of coupling into HOMs even when perfectly aligning the MFA with the NANF. As we mentioned, analysis of the optical spectrum of a broadband signal that propagates through NANF interconnected on both ends with SMF enables observation of coupling into HOMs. This manifests itself as an interference pattern. The light that is coupled into a HOM at the first MFA-NANF interface propagates in that mode and a fraction of it is coupled back into the fundamental mode of SMF after the second NANF-MFA interface. As the fundamental mode and the HOM have different effective refractive indices, the power recorded at the OSA shows a signature interference pattern. The amplitude of the interference pattern is proportional to the magnitude of the HOM excitation, while the interference period is proportional to the difference in propagation constants between the HOM and the fundamental mode of the NANF. Thus, we can distinguish between coupling to various HOMs too. This approach is significantly faster and easier to use than the traditional time of flight method^19^, which is especially of interest during the alignment of the interconnection, in which we try to simultaneously maximize the output power (hence coupling into the fundamental mode) and minimize the HOM interference pattern amplitude observed at the OSA (to minimize coupling into HOMs). It is important to note that in practice this technique is only possible with short pieces of fiber due to the limitation of the OSA resolution. E.g. our OSA (Yokogawa AQ6370C) with a minimum resolution of 0.02 nm allows the use of 44 m long fiber.

### Interconnection loss with GRIN MFAs

**Figure 5:**
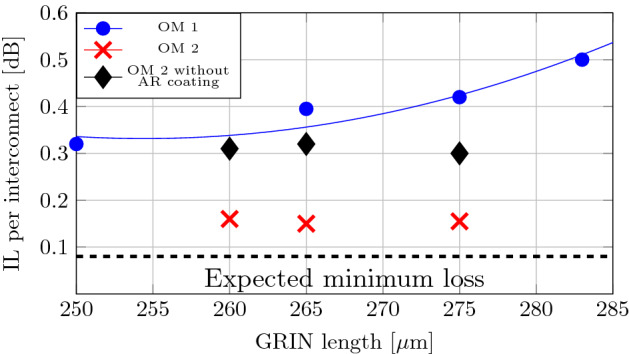
Insertion loss of SMF-28-NANF interconnect using OM1 (AR coated, blue circles, solid blue line represents fitted measurement data) and OM2 with (red crosses) and without (black diamonds) AR coating GRIN MFAs.

In this work we consider commercially available GRIN fibers to find the best MFD match. For our NANF we found the OM2 GRIN fiber to provide the closest match with the OM1 coming the second. However, depending on the particular NANF and its corresponding MFD, different type of GRIN fiber can provide an optimal mode-field adaptation. The insertion loss of SMF-NANF interconnections was measured for each GRIN MFA pair, see Fig. 5.

For OM1-based AR-coated MFA, a minimum loss of 0.32 dB was achieved with the GRIN fiber 1/4 pitch long ($250\,\upmu \hbox {m}$, Fig. 5), corresponding to the largest MFD that can be provided by the OM1 based MFA, Fig. 2. This MFD (19.1µm) is smaller than what is necessary for our NANF (Fig. 1d, MFD about $24\,\upmu \hbox {m}$). The expected loss is 0.35 dB (Fig. 2c), which is close to the measured loss of 0.32 dB. We speculate this small discrepancy is caused by the small gap between the GRIN MFA and NANF fiber that was made during the alignment targeted to find the best value, which may have slightly enlarged the beam MFD (an effect we have not accounted for in our theoretical analysis).

The OM2-based MFA is expected to give significantly better match of the mode field size (up to $23.2\,\upmu \hbox {m}$ in 1/4 pitch GRIN length, which is achieved for $300\,\upmu \hbox {m}$, Fig. 2a) to that of our NANF. As coupling between Gaussian fields of $23.2\,\upmu \hbox {m}$ and $24\,\upmu \hbox {m}$ MFDs leads to a negligible loss ($<0.01\,\hbox {dB}$, Fig. 2b), we expect the NANF-MFA interconnection loss to be dominated by the 0.08 dB additional loss due to the symmetry and shape mismatch. Our experiments show a loss only slightly higher than expected: 0.15 dB. This was achieved for a slightly shorter GRIN length $(265\,\upmu \hbox {m})$, which however is, similarly to the optimum 1/4 pitch length, expected to bring negligible (< 0.01 dB) mode field size mismatch loss, Fig. 2b. The 0.15 dB loss includes the loss of the SMF-GRIN splice. We measured this splice loss and found it to be below our power measurement resolution ($\le$ 0.01 dB). To evaluate the effect of the AR coating on the insertion loss, we also measured IL with the GRIN-MFAs before applying the coating, Fig. 5, showing 0.15 dB increase in the IL (corresponding to 3.5%), which is exactly in line with the value expected theoretically for the air-silica glass interface.

Unlike our previous study with photonic bandgap HCFs^9^ in which we concluded that the GRIN length in MFA is very critical ($\pm \,5\,\upmu \hbox {m}$ length change produced almost 0.1 dB interconnection loss increase), here we see that significantly larger GRIN length variation in here-used HCF and GRIN MFA should produce negligible interconnection increase (Fig. 2c). Specifically, a change of up to $\pm \,50\,\upmu \hbox {m}$ in GRIN length is predicted to produce insertion loss degradation below 0.01 dB. Experimentally, we confirm this, albeit over only a limited GRIN length variation of $15\,\upmu \hbox {m}$. This improvement is thanks to the MFA used in combination with the HCF which was designed to ensure that the MFD of the mode generated with the quarter-pitch length GRIN is well matched to the MFD of the NANF. The larger margin on the GRIN length we show here can be achieved with cleaving^20^, reducing the complexity of preparing an optimum-length GRIN MFA.

### Interconnection loss with TEC MFAs

**Figure 6:**
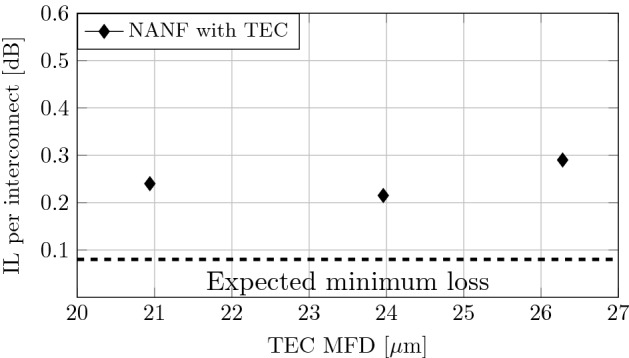
Insertion loss of SMF-28-NANF interconnect using TEC-based MFAs of various MFDs.

The insertion loss for each TEC MFA pair was measured, Fig. 6. As expected, the TEC producing a MFD of $24\,\upmu \hbox {m}$ gives the lowest insertion loss. We measured 0.21 dB, which is 0.13 dB above the mode symmetry and shape mismatch limit of 0.08 dB. For 21 and $26.5\,\upmu \hbox {m}$ MFD TECs, we measured an insertion loss of 0.24 and 0.29 dB, respectively. Based on the mode size mismatch, we would expect degradation of 0.08 dB for $21\,\upmu \hbox {m}$ and 0.04 dB for $26.5\,\upmu \hbox {m}$ as compared to the optimum $24\,\upmu \hbox {m}$. These very small values do not correlate well with the experiment (although they are of the same magnitude as measured). This can be caused either by mode shape mismatch (the TEC-MFA mode can deviate more from the Gaussian shape for larger MFDs) or by inner TEC insertion loss (larger TEC-MFA having higher insertion loss). Both of these are plausible, as larger MFD TEC expansion requires longer heat processing and thus provides less control over the shape of the output TEC refractive index and mode field profile.

### Measurement of higher-order mode content

**Figure 7:**
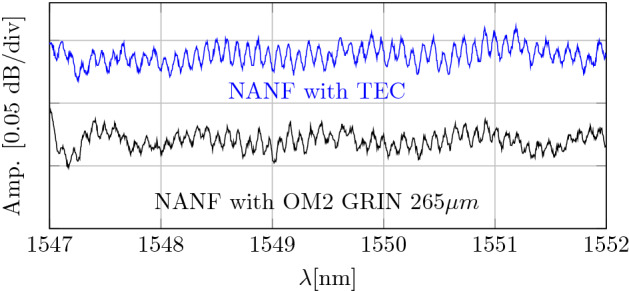
Interference pattern due to coupling into HOMs measured with the best-performing GRIN and TEC MFAs.

HOM interference patterns (optical spectrum measured at the SMF-MFA-NANF-MFA-SMF output) measured when using the best-performing pairs of GRIN and TEC MFAs are shown in Fig. 7.

**Figure 8:**
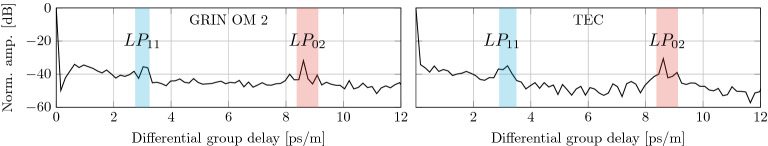
Fourier transform of spectral trances shown in Fig. 7. Positions of LP$_{11}$ and LP$_{02}$ modes expected from simulations are 3.1 and 8.7 ps/m.

We see very weak (below 0.05 dB peak-to-peak) spectral oscillations of the transmitted power. The period of the oscillation is directly proportional to the propagation delay in NANF between the fundamental mode and a HOM that is being excited. Thus, a Fourier transform of the spectrum enables us to analyze the HOMs that are being excited, as we know the guided modes propagation constants from simulations. Figure 8 shows the Fourier transform of data from Fig. 7, in which we have identified two HOMs that were predominantly excited: LP$_{11}$ (expected from simulations to be at 3.1 ps/m) and LP$_{02}$ (expected from simulations to be at 8.7 ps/m) modes. To estimate how much power is carried by these two modes, we have to find the relationship between the coupling efficiency for these HOMs and the measured quantities.

Considering only the fundamental mode and a single HOM, the detected power is:

1 $$\begin{aligned} |E + \alpha E\cos {\left( \Delta \tau L\right) }|^{2} = |E|^{2} + 2\alpha |E|^{2}\cos {(\Delta \tau ),} \end{aligned}$$

where $\alpha$ is the fraction of energy that couples into the HOM, propagates in the HOM, and then couples back into the fundamental mode. $\Delta \tau$ is the normalized differential delay between the two modes inside the NANF. *L* is the fiber length and *E* is the intensity of the electric field. We have neglected the term proportional to $\alpha ^2$. Then, the Fourier transform of the measured data is:

2 $$\begin{aligned} \mathscr {F}\left( |E + \alpha E|^{2}\right) = |E|^{2} \delta (t)+\alpha |E|^{2} \left( \delta (t-\Delta \tau ) + \delta (t+\Delta \tau )\right) . \end{aligned}$$

In Fig. 8, we see the tone at $t-\Delta \tau$ (due to the beating between the NANF fundamental mode and the HOM) which has a normalized amplitude of $\alpha$. When taking into account the calculated attenuation of the LP$_{11}$ and LP$_{02}$ modes mentioned earlier (we need to separate the $\alpha$ contributions due to attenuation and due to coupling at the MFA-NANF interfaces) and assuming the HOM coupling is identical at both MFA-NANF interfaces, we calculated that the coupling magnitude at a single GRIN-MFA interface into the LP$_{11}$ and LP$_{02}$ modes is −35.3 dB and −21.3 dB, respectively. The LP$_{11}$ mode has negligible loss for the 10-m long NANF sample used (35 dB/km), and thus the measured tone amplitude in Fig. 8 corresponds directly to the coupling into the LP$_{11}$ mode. However, the loss for LP$_{02}$ (2100 dB/km) cannot be neglected (21 dB in 10 m of NANF) and contributes significantly to the calculated coupling efficiency into the LP$_{02}$ mode. For TECs, the LP$_{11}$ coupling is very similar, −35.0 dB, but coupling into LP$_{02}$ is slightly higher at −20.1 dB.

The measured results for coupling into the LP$_{11}$ (−35 dB) and those simulated considering slight deviation from the perfect symmetry of the used NANF sample (−41  dB) are very close, especially considering measurement and simulation errors (due to limited resolution of the trace in Fig. 8 and limited accuracy of extracting data from the measured cross-sectional fiber image used in the simulations). In light of this, we conclude that coupling into the LP$_{11}$ mode at a single MFA-NANF interconnection is most likely limited by the symmetry in the fabricated NANF rather than by other effects such as poor cleave quality. This conclusion is further supported by the fact that we measured an almost identical level of LP$_{11}$ coupling using both, GRIN and TEC MFAs, suggesting this coupling is more related to the NANF properties rather than anything else. As for the LP$_{02}$ results, we measured slightly higher values than predicted (−24 dB), suggesting the mode field profile generated in MFAs deviates slightly from a Gaussian (as the simulations were based on an MFA with Gaussian mode field profile). This slight deviation may also explain why we have not achieved an insertion loss closer to the expected value of 0.08 dB (which is also calculated considering a Gaussian-profile input beam). Furthermore, in the GRIN-MFA, the measured value of −22.3 dB is closer to the prediction (−24 dB) than the value measured for a TEC-MFA (−20.1 dB), suggesting the mode field profile at the TEC output deviates more from the Gaussian than at the GRIN-MFA output. This may explain why we measured the insertion loss with the GRIN-MFA to be slightly smaller (0.15 dB) than for TEC-MFA (0.21 dB).

It is worth mentioning that a low-level of cross-coupling into LP$_{11}$ mode (e.g., −35 dB in our experiment) is more critical than coupling into other HOMs due to the relatively low LP$_{11}$ mode attenuation. The relatively high level of cross-talk into the LP$_{02}$ mode is then less critical for most applications due to the very high attenuation of the LP$_{02}$ mode in typical NANFs. Apart from the LP$_{11}$ mode, all HOMs suffer attenuation similar to or higher than LP$_{02}$ mode.

We conclude that the predominant loss mechanism of our interconnection is through coupling into the LP$_{02}$ mode. We expect this could be minimized by designing the GRIN fiber (to have a refractive index profile that slightly deviates from parabolic) or by optimizing the fiber (refractive index profile) used in the TEC process. As far as reduction of the LP$_{11}$ mode coupling concerns, this would require more symmetric structure of the fabricated NANF. Since better symmetry will also bring lower attenuation^2^, LP$_{11}$ mode coupling will be further reduced for lower-attenuation NANFs that have been already reported recently^2^.

### Permanent interconnection

Based on the results shown in Fig. 5 we have proceeded to creating a permanent interconnection using a technique described in detail in^9^. We used a slightly modified setup from Fig. 4, where we added fiber-array holders (Thorlabs HFA001) onto the 5-axis stages.

The GRIN MFA, as discussed above, is glued in a fiber array and then polished to the desired length and AR coated. NANF fiber array, however, is prepared slightly differently, because the end-face of the NANF cannot be polished, as debris would get into the holes. This modified procedure has three steps. Firstly, after striping the protective coating from the small portion of NANF, we cleaved it, and inserted it into an empty pre-polished fiber array. Then, the NANF cleaved end-face was aligned with the pre-polished fiber-array end-face, so that both NANF and fiber-array end at the same point. Finally, we applied a UV-curable glue to the back-side of the NANF fiber array to secure NANF inside it.

Afterwards, we put both fiber arrays (with GRIN MFA and NANF) into the fiber-array holders, which we fixed on our 5D stages (Thorlabs NanoMax MAX313D/M with pitch and yaw tilt platform APY002/M), enabling precise alignment in the same way that was used previously for characterization. After aligning the two fiber arrays, we applied a viscous UV-curable glue in between the two fiber arrays (ensuring the glue does not creep into the NANF microstructure) and cured the glue to form a permanent interconnection. During the curing process, we continuously monitored IL of the interconnection and observed an IL degradation of 0.01 dB, which is within our measurement accuracy. Thus, we conclude that the gluing did not degrade the interconnection performance.

It is worth mentioning that although we made the interconnections manually (as a proof of concept), the entire process can be in principle fully automated, as it is for conventional gluing-based fiber array assembly in the photonic industry.

## Conclusion

We have demonstrated record low-loss interconnect between NANF and SMF with insertion loss of only 0.15 dB. This is 0.07 dB above the theoretically-expected minimum loss due to the mode shape and symmetry mismatch between the NANF mode and an ideal Gaussian mode. The interconnect was based on a modified fiber-array technology, which is industry-proven, used, e.g., in telecom when attaching SMFs to high-port-number planar lightwave splitters (e.g., 1 $\times$ 64). As gluing is a cold process (no fusion splicing involved), it allows for deposition of optical coatings in between NANF and the SMF-GRIN mode field adapter. Anti-reflective coatings strongly-reduce the SMF-NANF 3.5% back-reflection due to the hollow core (air) to glass core interface. We experimentally demonstrated better than −40 dB back-reflection over 60 nm bandwidth while reducing the interconnection insertion loss by 0.15 dB (corresponding to the theoretically-expected reduction by 3.5%).

We used two approaches for adaptation of the mode field size between the SMF and NANF. The first mode field adapter used a short segment of telecom-standard (OM2) graded-index multimode fiber. The interconnection insertion loss was 0.15 dB, where we showed that a length variation as large as $15\,\upmu \hbox {m}$ (such accuracy can be straightforwardly achieved with a modified standard fiber cleaver) does not degrade the excellent interconnection insertion loss performance.

The second mode field adaptation approach used a thermally-expanded core of SMF. TECs are available commercially at low cost and can be manufactured with advanced commercially-available fusion splicers. We achieved NANF-SMF interconnection loss of 0.21 dB with TEC mode field adapters.

We studied the unwanted coupling into higher-order modes of NANF. We measured HOMs using a simple method based on an analysis of optical spectra. For the lowest-attenuation HOM (LP$_{11}$, about 35 dB/km in our NANF), we found unwanted coupling below −35 dB being most likely limited by the slight asymmetry of the fabricated NANF (rather than alignment or NANF end-face cleaving imperfection). The second most prominent HOM into which a small portion of light was coupled is the LP$_{02}$ mode (attenuation of 2100 dB/km in our NANF). Coupling into this mode, which has the same symmetry as the LP$_{01}$ mode, is mainly due to the mismatch of the shape of the mode field adapter output and the NANF LP$_{01}$ fundamental mode profile. We show that GRIN-based mode field adapter produces slightly smaller coupling into the LP$_{02}$ mode (−21.3 dB) than the TEC mode field adapter (−20.1 dB), suggesting the first one has a mode field shape better adapted to the LP$_{01}$ mode than the latter. This agrees with our insertion loss measurements, which show slightly worse result for TEC mode field adapters (0.21 dB versus 0.15 dB).

We then show that permanent glued interconnection of NANF with a GRIN-based mode field adapter brings negligible (0.01 dB in our measurement, which is at our resolution limit) degradation to the final interconnection.

This work represents a new benchmark in hollow core fiber interconnection, showing simultaneously low loss, low coupling into higher-order modes, and low level of back-reflection. It also outlines further necessary steps to improve this even further (e.g., by improving the symmetry of the hollow core fiber or engineering the mode field profile generated by mode field adapters to better match the shape of the hollow core fiber fundamental mode).
